# Supplementary material for: Non-Destructive Determination of Alkylresorcinol (ARs) Content on Wheat Seed Surfaces and Prediction of ARs Content in Whole-Grain Flour
Source: Molecules. 2019 Apr 4;24(7):1329. doi: 10.3390/molecules24071329 (PMC6479818; doi:10.3390/molecules24071329)
Supplement: Supplementary file 1 [file molecules-24-01329-s001.pdf]

Non-Destructive Determination of Alkylresorcinol (ARs) Content on Wheat Seed Surfaces and Prediction of ARs Content in Whole-Grain Flour

Jiahuan Wang, Xin Gao * and Zhonghua Wang *

State Key Laboratory of Crop Stress Biology for Arid Areas, College of Agronomy, Northwest A & F University, Yangling 712100, China; wangjiahuan327@126.com

* Correspondence: m18292459524@163.com (X.G.); zhonghuaawang@126.com (Z.-H.W.); Tel.: +86-29-87081538 (Z.-H.W.)

Table S1. Full name and molecular ions of n-alkylresorcinols and n-methylalkylresorcinols identified in whole grain and seed surface by GC-MS.

Abbreviation	Full name	Molecular ion[a]
ARs	n-alkylresorcinols	
AR 15:0	1,3-dihydroxy-5-pentadecylbenzene	464
AR 17:0	1,3-dihydroxy-5-heptadecylbenzene	492
AR 19:0	1,3-dihydroxy-5-nonadecylbenzene	520
AR 21:0	1,3-dihydroxy-5-heneicosylbenzene	548
AR 23:0	1,3-dihydroxy-5-tricosylbenzene	576
AR 25:0	1,3-dihydroxy-5-pentacosylbenzene	604
MARs	n-methylalkylresorcinols	
MAR 19:0	1,3-dihydroxy-2-methyl-5- nonadecylbenzene	534
MAR 21:0	1,3-dihydroxy-2-methyl-5- heneicosylbenzene	562
MAR 23:0	1,3-dihydroxy-2-methyl-5- tricosylbenzene	590
MAR 25:0	1,3-dihydroxy-2-methyl-5- pentacosylbenzene	618

[a]GC-MS electron impact ionisation molecular ion on the trimethylsilyl derivatised compound.

Table S2. Relative AR homologue composition on the seed surface from 36 wheat samples cultivated 2017 in China.

Samples	AR19:0	AR21:0	AR23:0	AR25:0
HWM6	7.18	27.78	40.48	24.55
YMM1	1.35	16.00	42.83	39.82
HH7	7.63	18.98	35.33	38.07
ZZM13	6.15	33.17	51.59	9.08
ZPM30	5.17	27.91	44.43	22.49
CX29	5.92	30.46	44.65	18.97
FDCM21	9.66	25.05	34.90	30.40
FDCM23	21.69	39.56	24.86	13.88
XR507	10.96	26.73	32.00	30.31
GM8	11.56	33.84	35.23	19.37
HC878	3.76	20.04	41.60	34.60
HZ166	5.09	25.71	36.93	32.27
PA0658	2.75	27.07	46.58	23.61
HDM981	5.63	30.29	40.53	23.56
LY3	6.00	27.32	42.10	24.57
ZM162	6.17	23.37	40.03	30.43
XM45	4.08	21.30	39.61	35.00
YM118	10.73	30.81	39.76	18.70
YM1	7.83	29.14	43.07	19.96
XN10	6.35	26.00	39.69	27.96
PX9	6.80	30.65	39.42	23.12
PM116	3.65	22.91	54.57	18.87
SM	6.31	29.16	41.59	22.94
CX216	4.32	25.85	43.32	26.52
ZY1	11.62	36.70	32.56	19.12
HM608	5.92	27.25	39.39	27.45
HH15173	2.31	23.86	47.36	26.46
XZ12	3.54	22.92	39.59	33.95
SM119	1.97	17.40	41.64	38.99
RQM32	6.20	24.74	39.76	29.31
XC998	4.07	20.10	39.81	36.02
XN923	3.46	26.38	45.89	24.27
TZS528	32.28	47.75	14.48	5.49
THM6	2.33	22.86	47.82	26.99
JM208	13.12	36.56	34.81	15.51
SC8658	10.91	33.45	35.56	20.08

Unit is %.

Table S3. Relative AR Homologue Composition in Whole Grain from 36 Wheat Samples Cultivated 2017 in China.

Samples	AR15:0	AR17:0	AR19:0	AR21:0	AR23:0	AR25:0
HWM6	1.59	5.76	36.52	45.88	7.74	2.52
YMM1	1.10	5.20	34.16	50.94	6.79	1.81
HH7	1.05	5.27	34.03	47.14	9.62	2.88
ZZM13	1.40	5.42	33.60	47.98	9.79	2.72
ZPM30	1.01	4.76	32.84	47.03	11.22	3.13
CX29	1.45	5.33	35.13	47.57	8.04	2.47
FDCM21	1.41	5.18	33.60	49.01	8.51	2.59
FDCM23	0.95	4.79	33.85	48.34	9.33	2.74
XR507	1.59	4.70	32.22	51.64	7.44	2.40
GM8	1.38	5.27	34.33	50.74	6.24	2.03
HC878	1.17	4.70	35.08	50.48	6.41	2.18
HZ166	1.98	4.61	32.62	49.16	8.29	3.35
PA0658	2.16	5.02	32.73	47.93	9.68	2.47
HDM981	1.17	4.40	34.00	48.48	9.07	2.87
LY3	1.05	4.94	33.45	46.13	10.74	3.69
ZM162	1.13	4.68	30.31	53.00	8.34	2.54
XM45	1.02	4.55	34.09	47.39	9.73	3.22
YM118	1.10	5.41	28.81	50.07	10.92	3.70
YM1	1.28	4.50	31.85	47.20	11.78	3.39
XN10	1.36	5.45	37.39	46.00	6.71	3.08
PX9	1.24	5.07	35.92	45.55	9.61	2.62
PM116	1.56	5.03	33.96	48.04	8.48	2.95
SM	1.15	5.02	35.03	48.70	8.00	2.10
CX216	1.44	4.75	29.07	47.35	12.86	4.53
ZY1	2.04	5.96	35.05	44.98	8.53	3.44
HM608	1.26	4.18	33.40	48.35	9.99	2.82
HH15173	1.55	4.23	30.19	52.02	9.54	2.47
XZ12	1.54	5.10	34.72	48.80	8.43	2.21
SM119	1.06	5.04	31.59	49.56	9.89	2.87
RQM32	1.43	4.77	35.69	46.56	9.14	2.40
XC998	0.93	4.79	36.36	47.90	7.91	2.11
XN923	1.45	5.33	35.13	47.57	8.04	2.47
TZS528	1.69	6.15	37.23	45.78	7.21	1.93
THM6	1.48	5.46	35.16	47.97	7.56	2.37
JM208	0.92	4.69	32.99	46.35	11.84	3.21
SC8658	0.89	4.11	34.76	48.19	9.23	2.83

Unit is %.
